# Soft Tissue Mass Extramedullary Plasmacytoma Following Radiation Therapy for Solitary Bone Plasmacytoma

**DOI:** 10.7759/cureus.43927

**Published:** 2023-08-22

**Authors:** Jong H Kim, Ray D Page, Yen-Chung Wang, Minh-Triet Nguyen, Kunal Elete

**Affiliations:** 1 Department of Internal Medicine, Medical City Weatherford, Weatherford, USA; 2 Department of Hematology and Medical Oncology, Center for Cancer and Blood Disorders, Fort Worth, USA; 3 Department of Internal Medicine, Medical City Fort Worth, Fort Worth, USA

**Keywords:** plasma cell tumor, plasma cell malignancy, plasma cell neoplasms, plasmacytoma treatment, solitary extramedullary plasmacytoma, solitary bone plasmacytoma

## Abstract

Soft tissue involvement in extramedullary plasmacytoma (EMP) is an exceptionally rare occurrence within the spectrum of plasma cell neoplasms. This case report presents the unique scenario of a patient who developed a soft tissue mass EMP subsequent to receiving radiation therapy for a solitary bone plasmacytoma at a distinct anatomical site. The primary objective of this report is to elucidate the clinical characteristics, diagnostic complexities, and management considerations associated with this uncommon presentation. Through a comprehensive review of existing literature, we aim to provide valuable insights and expertise to healthcare providers involved in the assessment and treatment of similar cases.

## Introduction

Extramedullary plasmacytoma (EMP) is a rare plasma cell neoplasm characterized by the proliferation of abnormal plasma cells outside the bone marrow. It represents approximately 3-5% of all plasma cell tumors and is often found in mucosal sites such as the upper respiratory tract or gastrointestinal tract [1,2]. Soft tissue involvement in EMP is an exceedingly uncommon manifestation, with only a limited number of cases reported in the medical literature [3]. This case report aims to present a unique case of soft tissue mass EMP that developed following radiation therapy for a solitary bone plasmacytoma at a different site.

The diagnosis and management of EMP pose significant challenges due to its rarity and varied clinical presentation. EMPs can occur as primary lesions or secondary to underlying plasma cell dyscrasias, such as multiple myeloma (MM) or solitary bone plasmacytoma (SBP) [1]. They are typically diagnosed in the sixth to seventh decade of life, with a slight male predominance [2]. However, soft tissue involvement further complicates the diagnostic process, as the symptoms and imaging findings can mimic other soft tissue tumors.

Radiation therapy is considered the standard treatment for localized EMP, providing excellent local control and favorable outcomes [4]. However, the development of a soft tissue mass EMP following radiation therapy for a solitary bone plasmacytoma adds an additional layer of complexity to the management of plasma cell neoplasms. Understanding the clinical characteristics, diagnostic challenges, and management strategies specific to soft tissue EMP is crucial for oncologists to provide optimal care for their patients.

In this case report, we present the clinical course of a 74-year-old male who initially presented with nasal pain, leading to the diagnosis of a left sinonasal plasmacytoma. The patient underwent radiation therapy in the fall of 2020 for the bone lesion, which resulted in complete resolution. However, the patient developed lower back pain and left leg weakness approximately 24 months later, ultimately diagnosed as soft tissue EMP. This case highlights the need for thorough evaluation and surveillance despite the successful treatment of bone plasmacytomas to detect and manage potential soft tissue involvement.

The objectives of this case report are to shed light on the rarity of soft tissue involvement in EMP, discuss the diagnostic challenges faced in identifying soft tissue EMP, and provide insights into the appropriate management strategies for this unique clinical presentation. By presenting this case and conducting a comprehensive literature review, we aim to enhance the knowledge and awareness among medical oncologists regarding this rare manifestation of plasma cell neoplasms, thus facilitating accurate diagnosis and tailored treatment approaches for similar cases in the future.

## Case presentation

**Figure 1:**
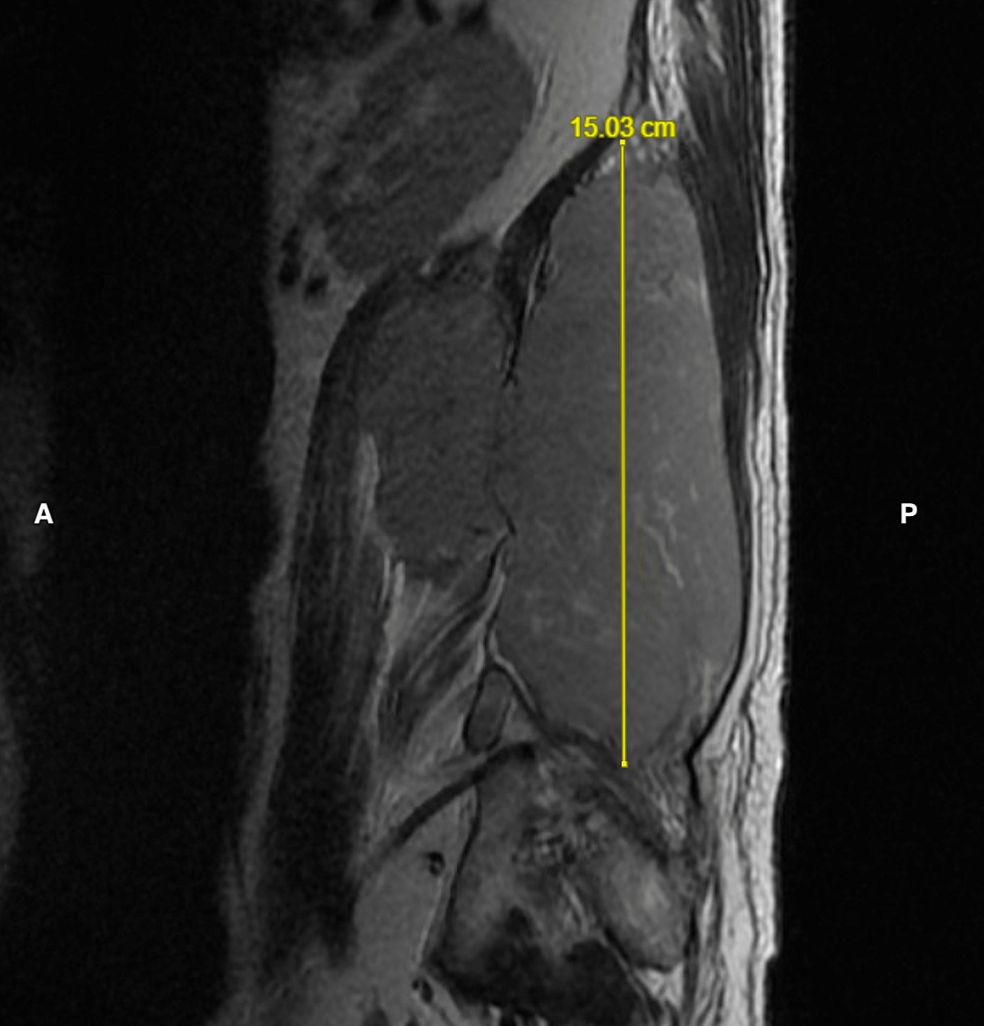
Soft tissue mass situated within the posterior side spanning approximately 15.03 cm craniocaudally in T2-weighted sagittal view. A = Anterior; P = Posterior

**Figure 2:**
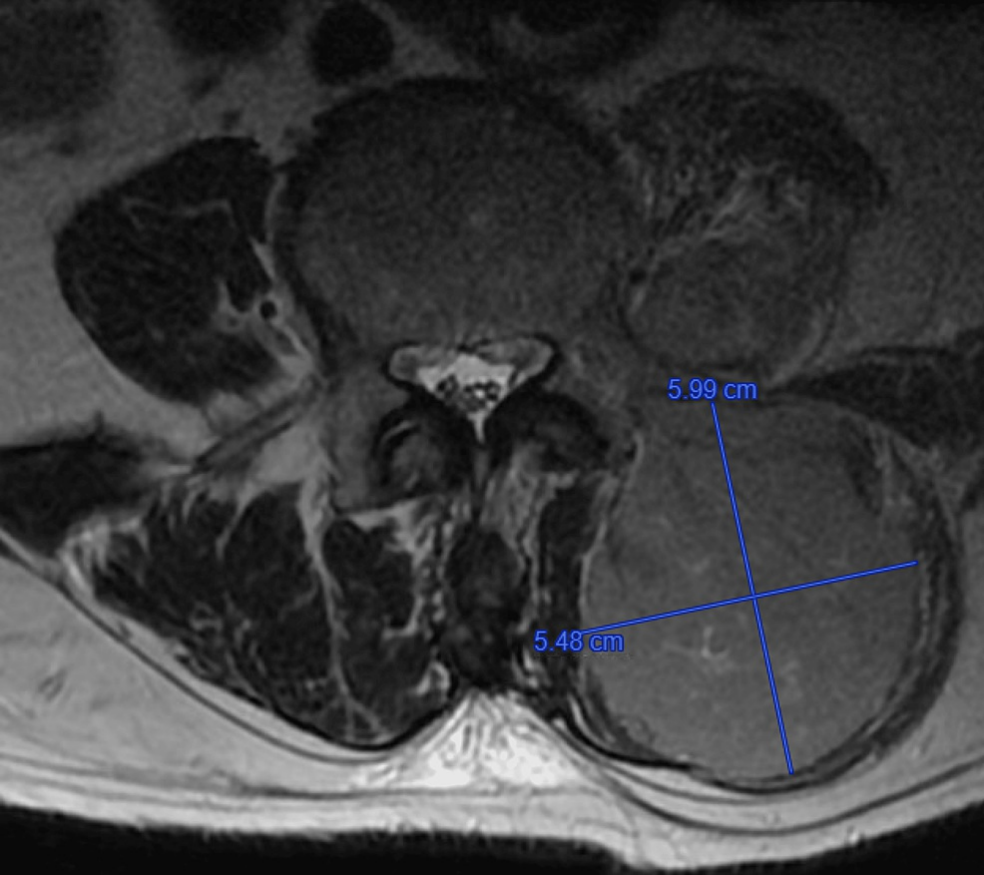
The larger constituent situated within the left posterior paraspinal muscle measuring 5.48 x 5.99 cm in T2-weighted axial plane view

**Figure 3:**
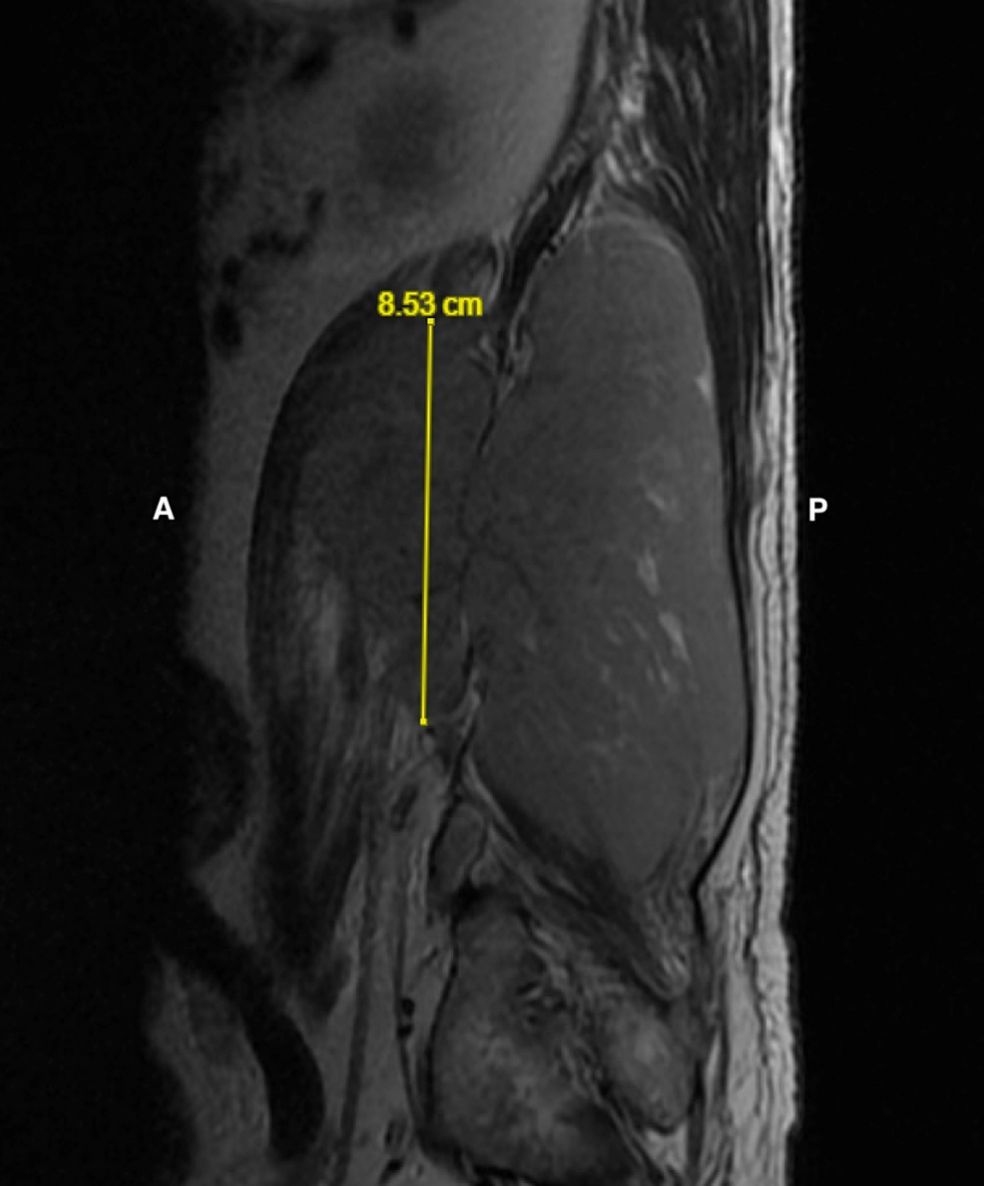
Anteriorly positioned paraspinous element extends roughly 8.53 cm craniocaudally in T2-weighted sagittal view A = Anterior; P = Posterior

**Figure 4:**
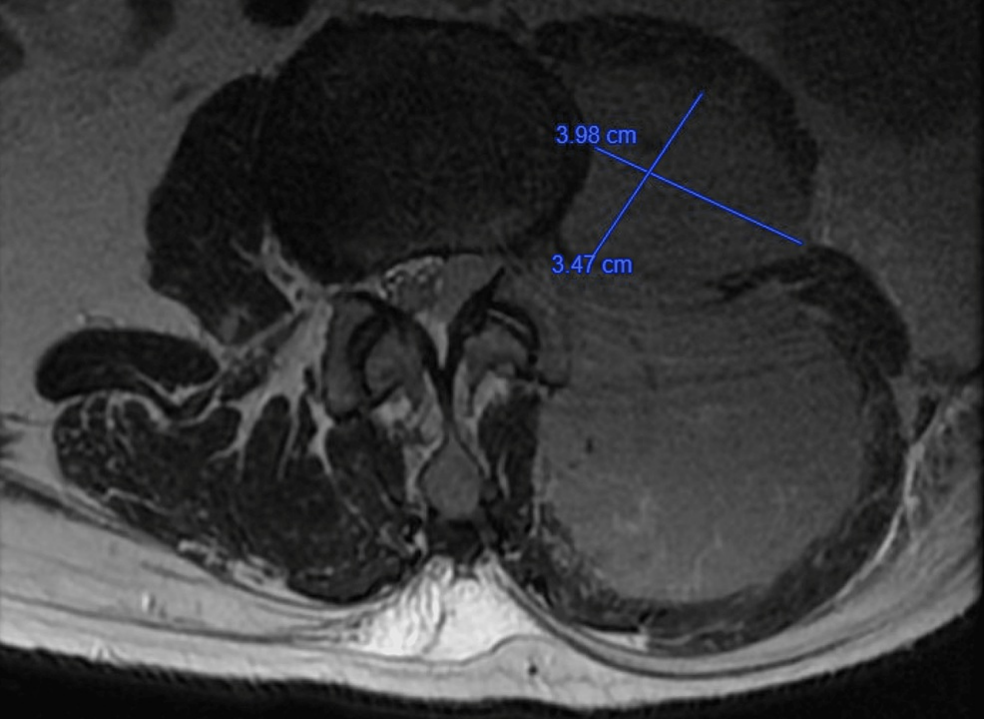
Anteriorly positioned paraspinous element measures 3.98 x 3.47 cm in the T2-weighted axial plane view

**Figure 5:**
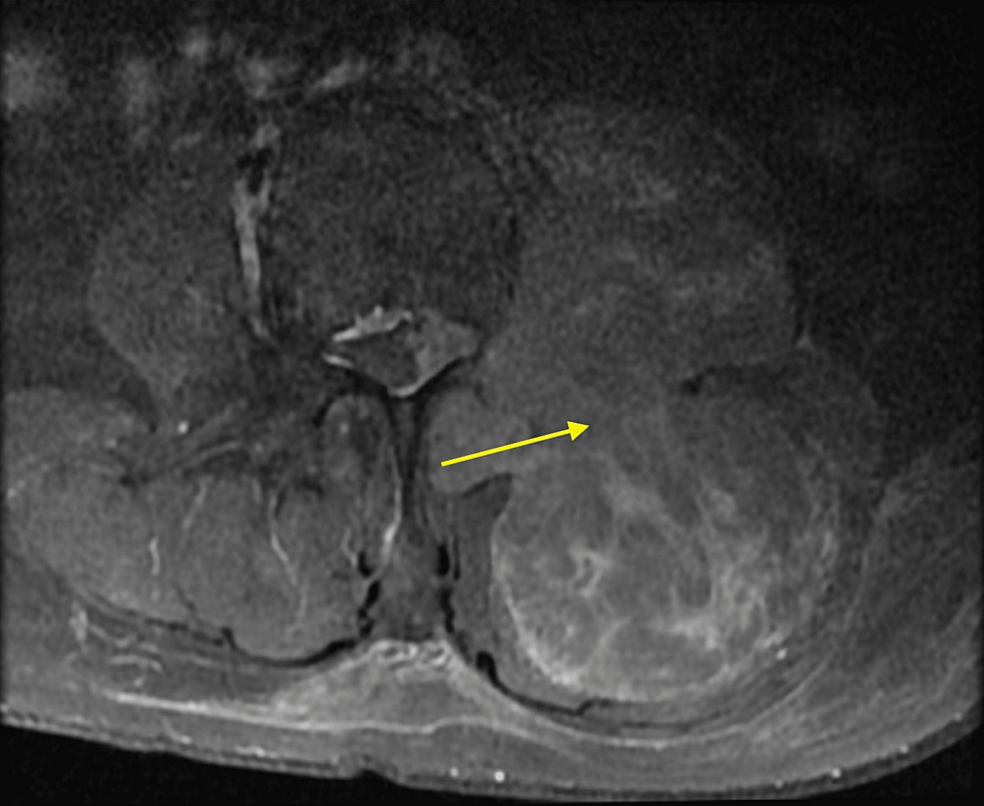
The interconnected anterior and posterior soft tissue components shown in the T1-weighted axial view with contrast

**Figure 6:**
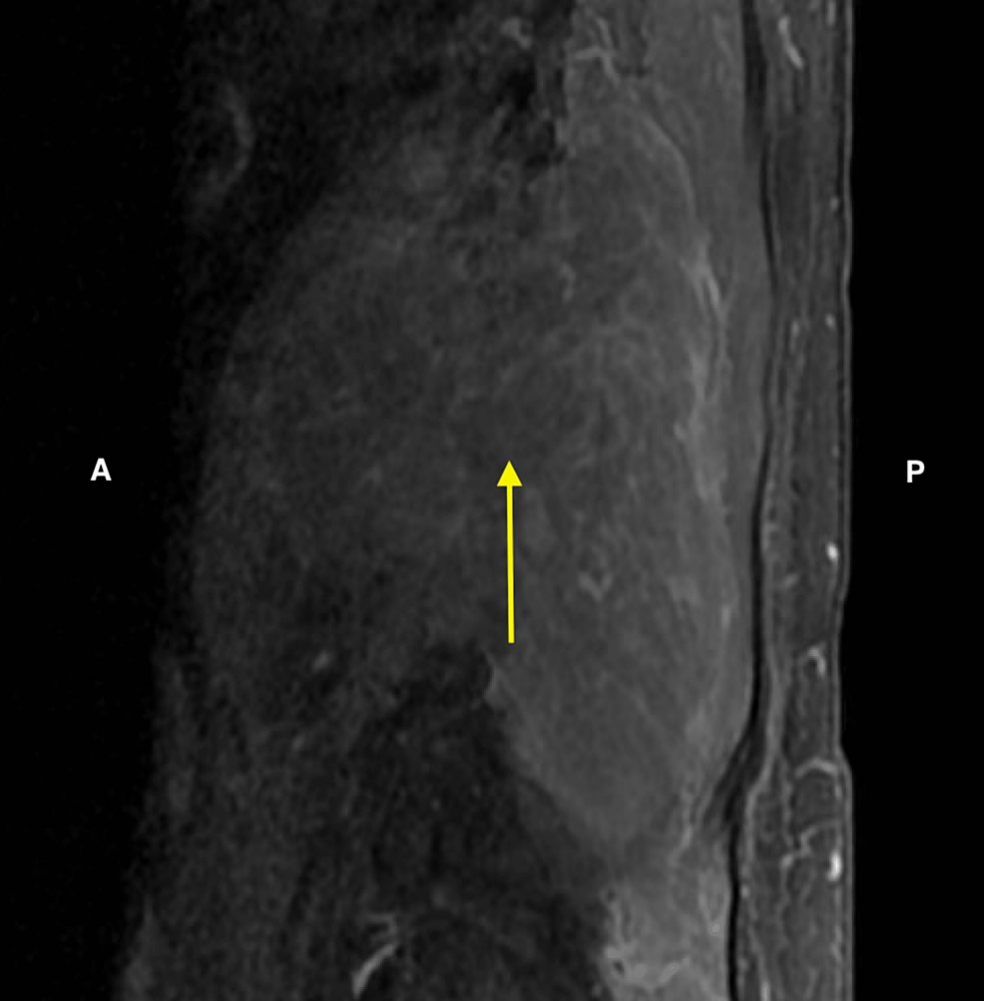
The interconnected anterior and posterior soft tissue components shown in the T1-weighted sagittal view with contrast A = Anterior; P = Posterior

**Figure 7:**
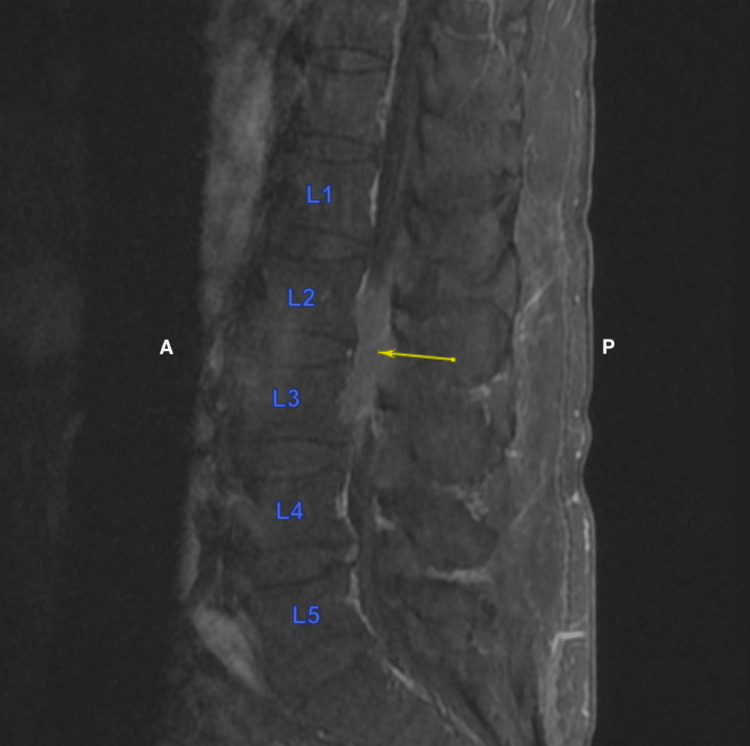
Extension of soft tissue in the vertebral canal and invasion into the vertebral body of L3 shown in the T1-weighted sagittal view with contrast A = Anterior; P = Posterior

**Figure 8:**
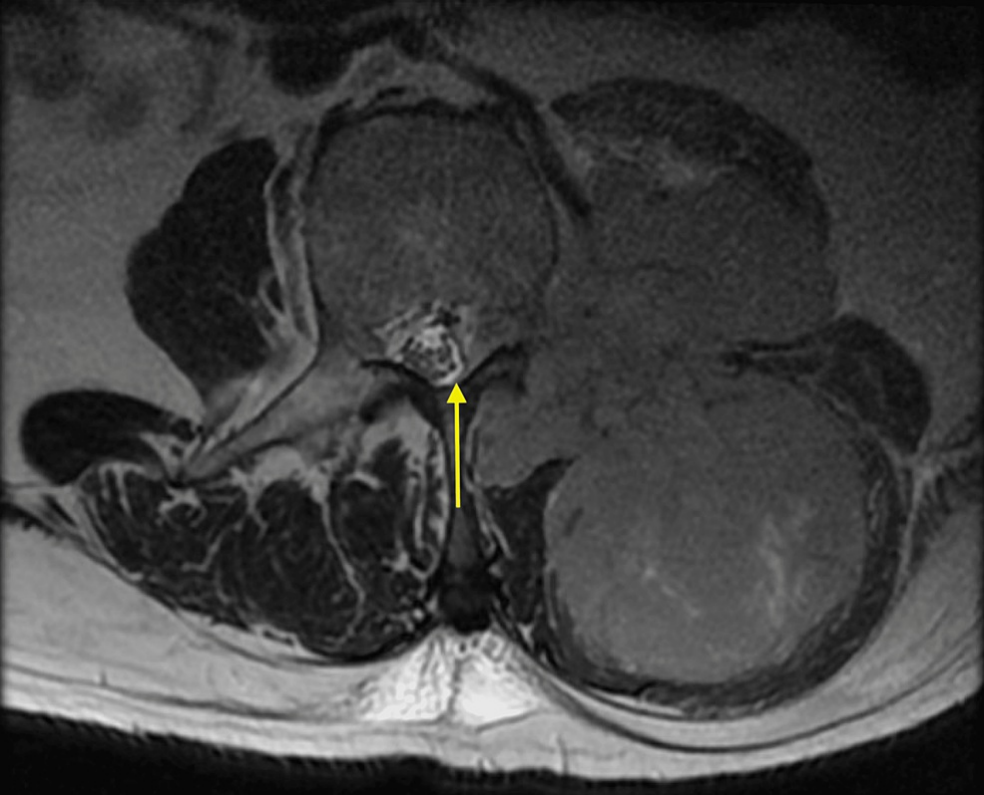
Soft tissue mass protruding into the leftward aspect of the central canal, causing displacement of the thecal sac to the right and resulting in a state of moderate central stenosis shown by the T2-weighted axial view

**Figure 9:**
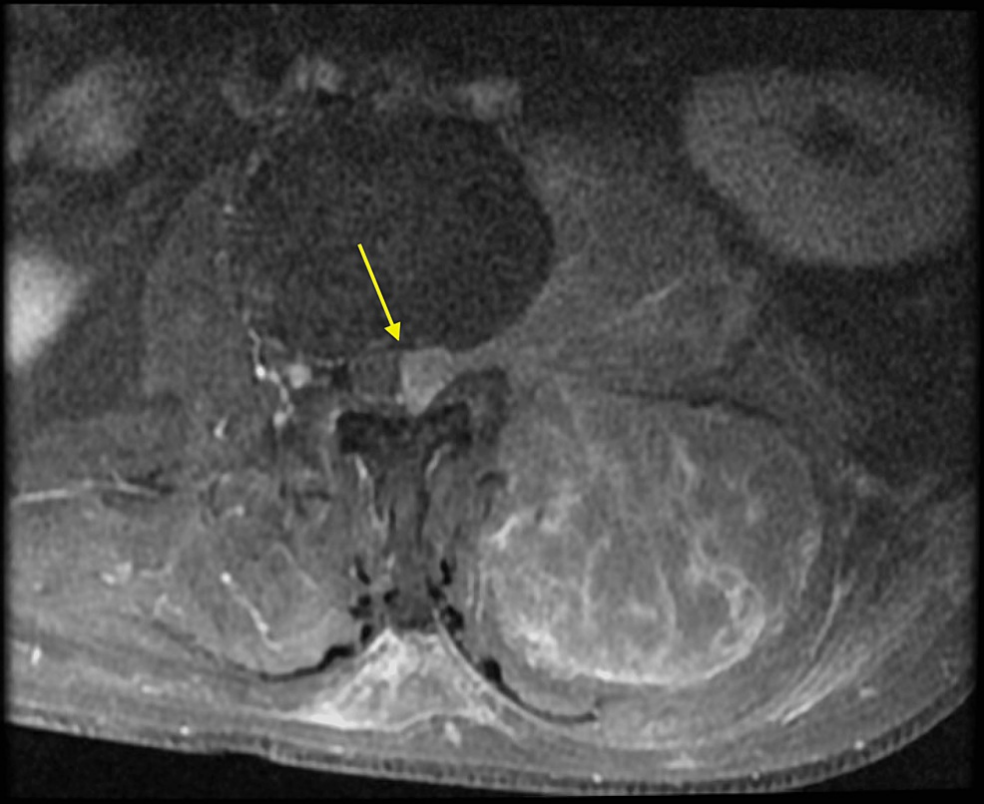
Protrusion of the soft tissue mass into the central canal shown by the T1-weighted axial view with contrast

A 74-year-old male with a past medical history of hypertension, hyperlipidemia, and isolated left sinonasal plasmacytoma status post radiation therapy without definitive information on dose or irradiation method, presented to the outpatient clinic with a three-week history of low back pain and left lower extremity weakness. The patient had received radiation therapy as a treatment modality for sinonasal plasmacytoma. Regrettably, subsequent to the intervention, the patient's trajectory was hindered by a lapse in follow-up. Subsequent to outpatient magnetic resonance imaging (MRI) with and without contrast, the presence of two substantial paraspinous components was detected as shown in Figures 1-6, as well as the invasion nature of the tumor into the central canal, as shown in Figures 7-9.

**Figure 10:**
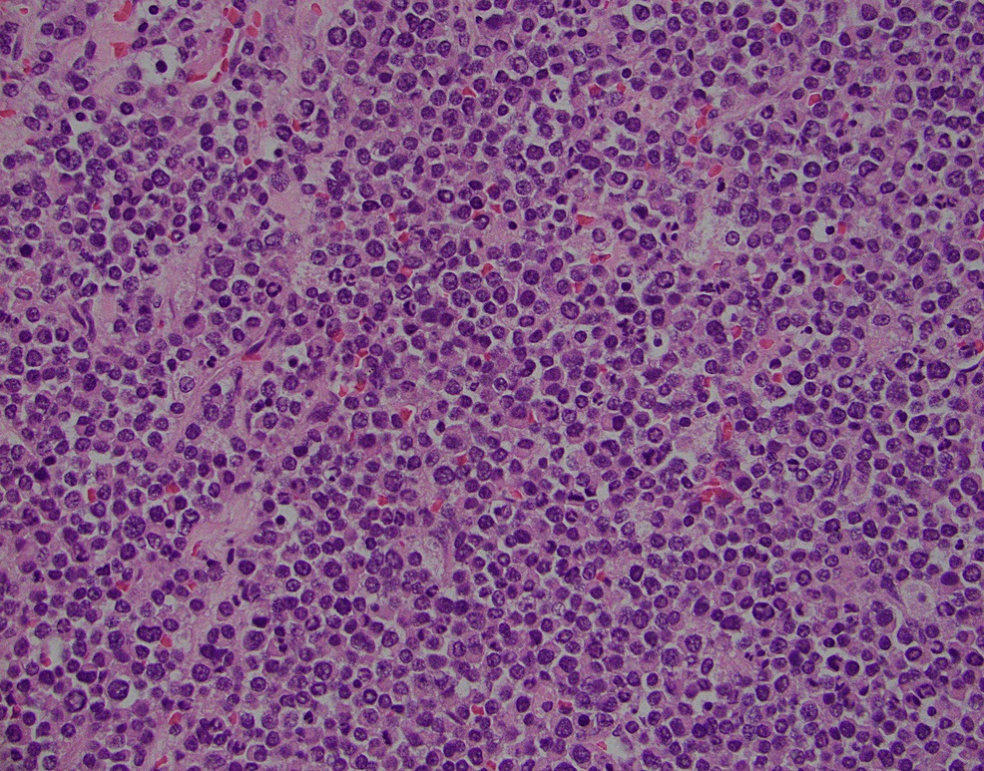
H&E stained slide of the lumbar mass depicting the extensive plasma cell infiltration of the tissue H&E = Hematoxylin + Eosin

A CT-guided biopsy of the mass was performed, which showed a proliferation of plasma cells, as shown in Figure 10.

**Figure 11:**
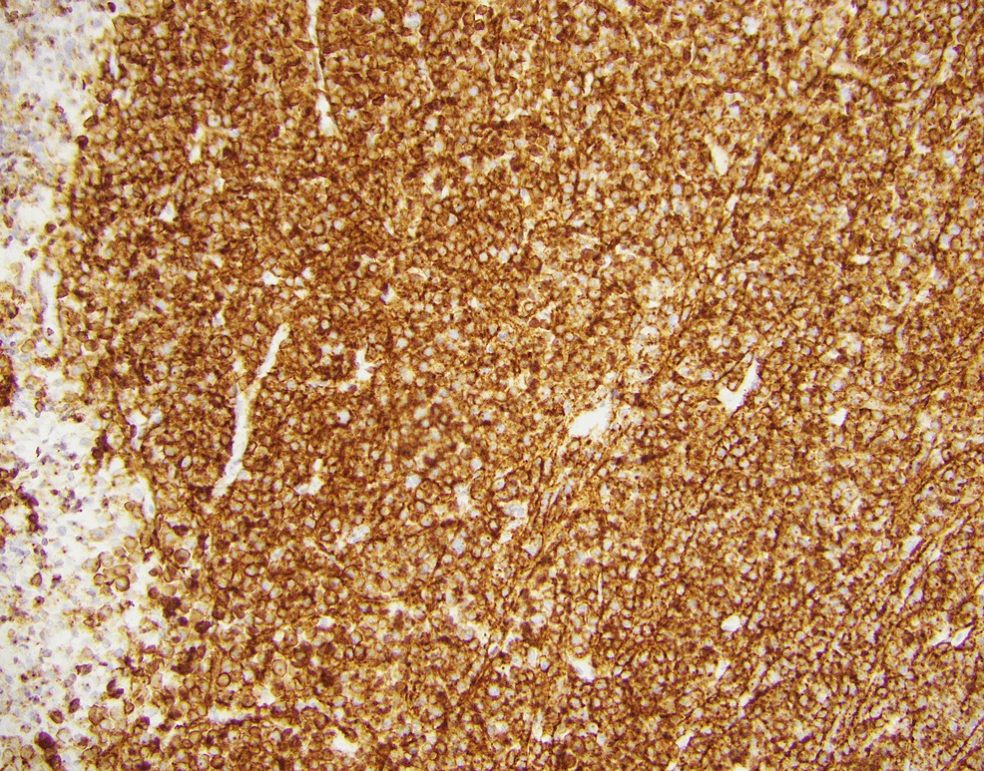
Diffuse positivity with CD138 immunostaining on the soft tissue biopsy CD138: Syndecan-1

The patient then presented to the emergency department a few days after the biopsy due to worsening intractable back pain and was admitted for suspected cord compression. The patient described the pain as being sharp and radiating down the left leg, unchanging with position, and not associated with urinary symptoms or sensory disturbances. Neurosurgery service was consulted and they recommended to follow up on the biopsy results, clearing the patient for discharge afterward, as the patient did not exhibit any neurological symptoms with consideration for radiation therapy. The biopsy later showed positivity with CD138 immunostaining, consistent with a plasma cell neoplasm, as depicted in Figure 11.

After his discharge from the hospital, the patient's pain and deficits in neurological symptoms regressed, prompting his return to the emergency room after three days. In response, the expertise of surgical oncology was consulted, collaborating in conjunction with the medical oncology services of the hospital. This collaborative effort led to the recommendation of administering pulse steroids with intravenous daily dexamethasone and radiation therapy. After thorough deliberation, both surgical oncology and neurosurgery concurred on pursuing a joint surgical debulking strategy to address the tumor, driven by the severity and rapid progression of the patient's symptoms. This was to be succeeded by a regimen of outpatient radiation therapy.

The subsequent surgical intervention encompassed an extensive resection of the left paraspinous mass, inclusive of muscle group resection and retroperitoneal resection. This was succeeded by a targeted L3 lumbar hemilaminectomy and medial facetectomy. Regrettably, a few hours following the procedure, the patient encountered a substantial episode of postoperative bleeding, necessitating an emergent return to the operating room. The ensuing exploration of the surgical cavity uncovered a hematoma within the left retroperitoneal space, stemming from apparent bleeding to the patient's laminectomy site. Hematoma evacuation and comprehensive washout were performed. Consequently, the patient was admitted to the neurosurgical intensive care unit for closer monitoring and care.

Over the ensuing days, the patient's recovery progressed favorably, marked by a notable absence of ongoing bleeding or leg pain. This recuperative trend prompted his eventual discharge, supplemented with home health care support for outpatient follow-up and radiation therapy as recommended by a radiation oncologist.

## Discussion

Soft tissue involvement in EMP is an exceptionally rare phenomenon, with only a limited number of reported cases. This manifestation represents a more aggressive form of plasma cell neoplasms, with an estimated incidence ranging from 1.7% to 4.5% [2]. The underlying etiology of EMP remains poorly understood; however, current evidence suggests that it arises from localized clonal proliferation of plasma cells. Hughes et al. conducted a study indicating differences in cellular adhesion molecule or chemokine receptor expression profiles may contribute to the development of EMP [5].

The occurrence of a soft tissue mass EMP subsequent to radiation therapy for a solitary bone plasmacytoma introduces a unique aspect to the clinical course of plasma cell neoplasms. This unusual presentation necessitates careful consideration by medical oncologists, as it poses several important challenges and management considerations. Heller et al. conducted a data analysis encompassing a literature review spanning the years 1998 to 2021, focusing on the anatomical distribution of EMPs. The findings of this analysis revealed a prevailing localization of EMPs in the upper aero-digestive (UAD) tract, accounting for 62.4% of cases, while the remaining 37.6% were situated in non-UAD regions. Among the non-UAD localizations, the gastrointestinal tract accounted for 31%, the lung region for 8%, the urogenital tract for 9%, and other non-UAD areas constituted 52%. It is noteworthy that the dataset did not explicitly address soft tissue localizations, likely due to their rarity; however, it can be inferred that soft tissue EMPs might exhibit a distribution similar to EMPs in general [2].

The diagnosis of soft tissue EMP poses a challenge due to its rarity and nonspecific clinical presentation. Differentiating it from other benign or malignant soft tissue tumors requires a high index of suspicion, particularly in patients with a history of plasma cell neoplasms. To evaluate the extent of disease involvement and guide treatment decisions, imaging modalities, such as magnetic resonance imaging (MRI) and positron emission tomography-computed tomography (PET-CT), are crucial. Histopathological examination remains the gold standard for confirming the diagnosis of soft tissue EMP. Fine-needle aspiration cytology or core needle biopsy can provide samples for evaluation. The histological features of soft tissue EMP include a proliferation of mature plasma cells with eccentric nuclei, abundant cytoplasm, and distinct perinuclear hofs. Immunohistochemical staining plays a vital role in confirming the plasma cell origin and excluding other potential differential diagnoses such as lymphoma or metastatic carcinoma [6].

Additionally, SBPs and solitary extramedullary plasmacytomas (SEPs) are distinguished from MM by the lack of the following criteria: anemia, hypercalcemia, renal insufficiency, or lytic bone lesions that may be attributed to MM. Additionally, diagnosis also requires bone marrow biopsy and immunoglobulin/light chain evaluation, to rule out MM [7]. Furthermore, although there is generally no involvement of the bone marrow, plasmacytoma may still be diagnosed if marrow involvement shows less than 10% of plasma clonal cells [6]. Both conditions have an increased risk of progression to MM despite appropriate radiation therapy. However, in certain retrospective studies, only 2% of patients with SBP have been found to develop new sequential plasmacytomas, as seen in this patient [8-10].

The management of soft tissue EMP necessitates a multimodal approach, which is tailored to the specific characteristics of the individual patient and the extent of the disease. When feasible, complete surgical resection is often recommended, as it improves local control and may potentially enhance patient outcomes [11]. Radiation therapy plays a crucial role in achieving local disease control and may be administered in various treatment modalities, including preoperative, postoperative, or definitive therapy for unresectable cases. However, due to the rarity of soft tissue EMP, the optimal radiation dose and field size have not been well-established [12]. Chemotherapy, particularly agents targeting plasma cells, such as bortezomib, can be considered in cases with systemic involvement or high-risk features [13]. However, the role of chemotherapy in the management of localized soft tissue EMP is less well-defined. Nevertheless, it is an appropriate consideration in those patients with suspicion of residual disease [14].

The prognosis of soft tissue EMP varies, with reported five-year survival rates ranging from 60% to 80%. Both SBP and SEP have similar median survivals, with SBP at 10 years and SEP at 11 years. Several factors can influence the prognosis of soft tissue EMP, including advanced age, high-grade disease, extensive soft tissue involvement, and systemic dissemination. These factors should be considered when assessing the patient's overall prognosis and treatment plan. Long-term follow-up is crucial in soft tissue EMP to monitor for disease recurrence or progression and to initiate appropriate interventions promptly if necessary. Regular clinical evaluations, radiological assessments, such as MRI or PET-CT, and laboratory tests are vital components of the follow-up protocol to detect any potential changes in disease status and ensure timely intervention [2].

## Conclusions

Soft tissue involvement in EMP is an exceptionally rare manifestation within the spectrum of plasma cell neoplasms. This case report has shed light on the unique scenario of a patient who developed a soft tissue mass EMP subsequent to radiation therapy for a solitary bone plasmacytoma at a different anatomical site. Diagnosing soft tissue EMP can be challenging due to its rarity and nonspecific clinical features. Therefore, maintaining a high index of suspicion is crucial, especially in patients with a history of plasma cell neoplasms. Radiological imaging along with histopathological examination play essential roles in confirming the diagnosis and guiding treatment decisions.

Long-term follow-up is crucial in monitoring for disease recurrence or progression, as soft tissue EMP can have variable prognoses. Regular clinical and radiological assessments, coupled with appropriate laboratory investigations, aid in the early detection of potential complications and the prompt initiation of necessary interventions. This case report highlights the need for further research and collaboration to enhance our understanding of the etiology, pathogenesis, optimal treatment approaches, and prognostic factors specific to soft tissue EMP. By presenting this case and reviewing the available literature, we aim to contribute to the collective knowledge of medical oncologists and provide valuable insights for managing similar cases in the future.
